# Blood pressure decreases after revascularization in atherosclerotic renal artery disease: A cohort study based on a multidisciplinary meeting

**DOI:** 10.1371/journal.pone.0218788

**Published:** 2019-06-24

**Authors:** Florence Sens, Gabrielle Normand, Thomas Fournier, Nellie Della-Schiava, Stéphane Luong, Caroline Pelletier, Philip Robinson, Sandrine Lemoine, Olivier Rouvière, Laurent Juillard

**Affiliations:** 1 Department of Nephrology, Edouard Herriot Hospital, Hospices Civils de Lyon, Lyon, France; 2 Lyon 1 Claude Bernard University, Villeurbanne, France; 3 FCRIN INI-CRCT, Nancy, France; 4 Department of Vascular Surgery, Edouard Herriot Hospital, Hospices Civils de Lyon, Lyon, France; 5 Department of Urinary and Vascular Imaging, Edouard Herriot Hospital, Hospices Civils de Lyon, Lyon, France; 6 Direction de la Recherche Clinique et de l’Innovation, Hospices Civils de Lyon, Lyon, France; International University of Health and Welfare, School of Medicine, JAPAN

## Abstract

**Background:**

In atherosclerotic renal artery disease, the benefit of revascularization is controversial. A clinical decision-making process based on a multidisciplinary meeting was formalized in the Lyon university hospital.

**Objectives:**

To investigate whether this decisional process ensured a clinical benefit to patients assigned to renal revascularization.

**Methods:**

Single-centre retrospective cohort study, including patients diagnosed from April 2013 to February 2015 with an atherosclerotic renal artery disease with a peak systolic velocity >180cm/s. For each patient, the decision taken in multidisciplinary meeting (medical treatment or revacularization) was compared to the one guided by international guidelines. Blood pressure values, number of antihypertensive medications, presence of an uncontrolled or resistant hypertension, and glomerular filtration rate at one-year follow-up were compared to baseline values. Safety data were collected.

**Results:**

Forty-nine patients were included: 26 (53%) were assigned to a medical treatment and 23 (47%) to a renal revascularization. Therapeutic decision was in accordance with the 2013 American Health Association guidelines and with the 2017 European Society of Cardiology guidelines for 78% and 22% of patients who underwent revascularization, respectively. Patients assigned to revascularization presented a significant decrease in systolic blood pressure (-23±34mmHg, *p* = 0.007), diastolic blood pressure (-12±18mmHg, *p* = 0.007), number of antihypertensive medications (-1.00±1.03, *p* = 0.001), and number of uncontrolled or resistant hypertension (*p* = 0.022 and 0.031) at one-year follow-up. Those parameters were not modified among patients assigned to medical treatment alone. There was no grade 3 adverse event.

**Conclusion:**

Based on a multidisciplinary selection of revascularization indications, patients on whom a renal revascularization was performed exhibited a significant improvement of blood pressure control parameters with no severe adverse events.

## Introduction

Atherosclerotic renal artery disease represents a frequent and severe medical condition, the treatment of which remains controversial. Options for atherosclerotic renal artery disease treatment are medical therapy alone or medical therapy combined with renal artery revascularization, either with an endovascular technique or during an open surgery.

Currently, the benefit of revascularization has been challenged since three large randomized controlled trials failed to demonstrate any improvement in clinical outcomes after endovascular revascularization compared to medical therapy [1–3]. However, patients selection in these trials raises concern as enrolled patients presented mostly with a moderate degree of stenosis (50 to 70% diameter reduction), a moderately uncontrolled hypertension, a relatively stable kidney function, and did not experienced symptoms such as pulmonary oedema [4]. Furthermore, whereas renal artery disease is a relatively common condition, both the CORAL and ASTRAL trials required substantial protocol changes during enrolment to reach their recruitment goals [5]. Given the restricted population included in these randomized trials, to conclude that renal revascularization is of no benefit to any patient with atherosclerotic renal artery disease remains controversial [6–8].

To deal with the lack of valid scientific data applicable to all patients, a multidisciplinary renal artery disease meeting has been conducted every two weeks in a French university hospital starting from April 2013. We hypothesized that a multidisciplinary and individualized selection of revascularization decisions in atherosclerotic renal artery disease patients could ensure a clinical benefit to revascularized patients. Herein, we described the clinical decision-making process, and analysed whether patients who benefited from a revascularization exhibited clinical improvement.

## Materials and methods

### Setting, study design and population

In accordance with the recommendations established in 2011 by the European Society of Cardiology (2011 ESC Guidelines) [9] and in 2013 by the American Health Association (2013 AHA Guidelines) [10], patients in our hospital presenting with clinical findings suggestive of a diagnosis of renal artery disease were evaluated to identify a potential renal artery disease [9,10]. Doppler ultrasound was the first-line screening modality [9]. The diagnosis of renal artery disease was established in case of a peak systolic velocity >180cm/s in the main renal artery [11].

Patients diagnosed with a renal artery disease had their charts reviewed during a multidisciplinary meeting. For the present study, patients whose chart had been reviewed between April 2013 and February 2015 were included. Patients with a fibromuscular dysplasia were excluded.

### Baseline data and follow-up

For the present study, medical records and meeting reports were analysed retrospectively. Uncontrolled hypertension was defined as a systolic and/or a diastolic measurement above 140 or 90mmHg, respectively. Resistant hypertension was defined as uncontrolled hypertension despite three antihypertensive medications belonging to three different drug classes. Estimated glomerular filtration rate (eGFR) was calculated based on the Chronic Kidney Disease-Epidemiology Collaboration (CKD-epi) formula. Worsening of renal function was defined as a decrease in eGFR ≥20% as compared to its baseline value. For patients with a bilateral disease, the radiological parameters related to the side with the highest peak systolic velocity were recorded.

For each patient, the decision taken during the multidisciplinary meeting (medical treatment or revacularization) was collected and compared to the one guided by international guidelines, that is to the one that would have been taken if the 2011 ESC [9], 2013 AHA [10] and 2017 ESC [12] Guidelines had been followed. Three situations were considered: 1/ revascularization was recommended (guideline class I to IIa), 2/ revascularization could be considered (guideline class IIb), and 3/ revascularization was not recommended or suggested. Patients with a clinical situation corresponding to a Class I, IIa or IIb recommendation were considered as eligible to be revascularized. The guidelines were considered as not followed when a revascularization was either performed but not recommended, or not performed but recommended with a I or IIa class guideline.

After a one year follow-up, the following data were collected: systolic and diastolic blood pressure, number of concomitant anti-hypertensive drug classes, eGFR and/or initiation of a dialysis treatment, proteinuria, survival status, grade 3 complications related to the procedure, and procedure failures.

### Statistical analysis

Continuous data were reported as mean ± standard deviation (SD). Categorical data were reported as count and percentage. Univariate inter-group comparisons were performed using Fischer’s exact test or Chi^2^ test for categorical variables, and Student’s t-test or Mann-Whitney-Wilcoxon for continuous variables. In each treatment group, parameters at one year were compared to pre-meeting data: continuous parameters were compared using paired-t-tests or Wilcoxon signed rank test for paired samples; categorical parameters using McNemars’ tests. Intergroup comparisons of the data relating patient evolution during follow-up was not performed owing to the existence of selection biases between groups. As previous studies reported negative results, we could not calculate a prospective or a priori power. A two-sided P-value <0.05 was considered statistically significant. Analyses were performed using Statistical Package for the Social Sciences (SPSS) version 19.0 (IBM software, USA).

### Ethics statement

This study agrees to the Principles of Helsinki Declaration. It was approved by the Institutional Review Board. Written informed consent did not apply because of the observational and retrospective design of the study. Data were anonymized prior to analysis. The use of these data was authorized and registered with the national data protection commission (Commission Nationale de l’Informatique et des Libertés, CNIL; authorization number 14–82).

## Results

### Conduct and description of the multidisciplinary meeting

The meeting took place twice a month. The contribution of at least one physician of each of the following core disciplines was necessary: an interventional radiologist, a vascular surgeon, an angiologist, and a nephrologist.

The discussion of each patient’s chart was conducted using a systematic approach. 1/ The physician in charge of the patient reported his/her history, comorbidities, clinical aspects (including blood pressure, glomerular filtration rate, and cardiac status) and the patient’s wishes or opinion regarding treatment. 2/ A radiologist presented and commented the patient’s radiological exams. 3/ The referent physician listed the arguments for and against a revascularization among a list established collectively. Arguments favouring revascularization included a resistant hypertension, an unexplained worsening of renal function, an unexplained sudden pulmonary oedema [9,10], an unexplained smaller kidney (kidney length 8–10 cm) [13], a solitary functioning kidney or a bilateral disease [10], and one or more radiological arguments to consider the stenosis as being >70% or having a hemodynamic impact on parenchymatous vascularization (asymmetry of the intra-renal resistive index >8%, acceleration time greater than 70ms, peak systolic velocity >320cm/s [14,15], loss of signal on magnetic resonance angiography, or luminal diameter reduction >70% on computed tomography angiography). Arguments against a revascularization were an age greater than 85 years, an altered general health status, a stage 5 chronic kidney disease and an atrophic kidney (kidney length <8cm). 4/ Each of the participating physicians gave his/her opinion regarding the benefit/risk balance of a revascularization and a discussion was conducted. If no consensual decision was obtained, a supplementary imaging exam was planned (mostly computed tomography angiography for stenosis severity and aortic and anatomic evaluations, and magnetic resonance angiography for stenosis severity evaluation). Patient case was then discussed yet again at the following meeting. 5/ If a decision of revascularization was taken, an endovascular approach was most often privileged; an open surgery was considered only in cases of indications of an associated surgical repair of the aorta [9] or severe atherosclerotic lesions of the arterial wall on computed tomography considered at an increased risk of atheroembolism. 6/ The chairperson formalized a report available in the patient’s medical chart.

### Medical treatment and procedural details

According to current standards [16], medical treatment was optimized for all patients when necessary with antiplatelet agents, statins, and antihypertensive treatment optimisation to reach blood pressure targets [17–19]. Patient follow-up did not differ depending on therapeutic decision, with at least a biannual visit.

Percutaneous endovascular revascularization was performed by senior interventional radiologists or senior vascular surgeons. The procedures were performed through a femoral approach unless the angle between the aorta and the renal artery needed a humeral approach. A 6-F introducer sheath was first placed in the aorta. A standardised endoarterial bolus of 2,500UI of unfractioned heparin was administered. A 0.018-inch guide wire was inserted through the catheter. The stenosis was pre-dilated with a 5-6mm balloon (Sterling monorail, Boston Scientific, Marlborough, MA, USA). Then a 5-6mm balloon-expandable stent (Tsunami, Terumo) was deployed. The length of the stent was adapted to the length of the stenosis.

### Patient characteristics

**Fig 1:**
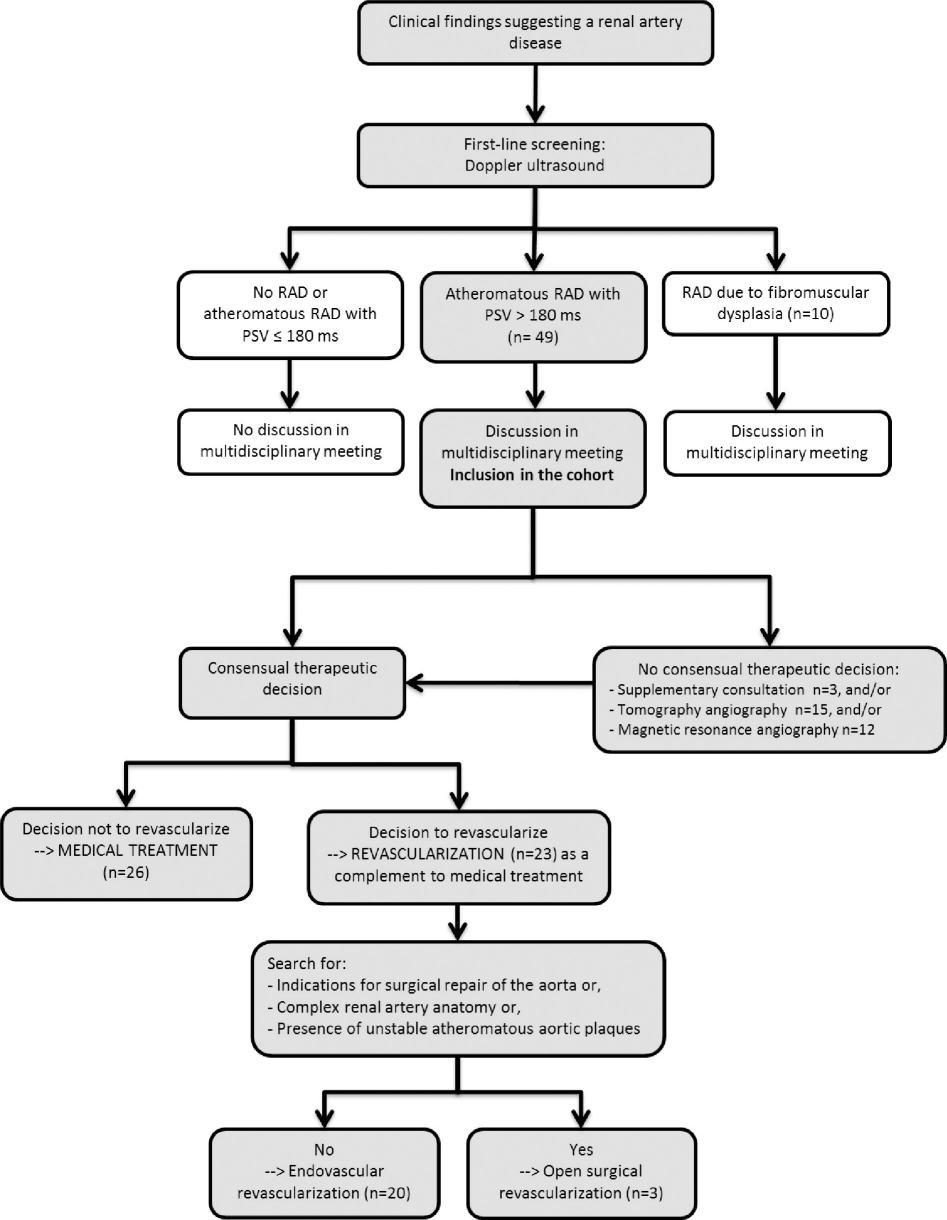
Flowchart. PSV: Peak Systolic Velocity; RAD: Renal Artery Disease.

Forty-nine patients were included (Fig 1). Baseline characteristics are provided in Table 1. Overall, 59% of patients presented with an uncontrolled hypertension, and 24% with a resistant hypertension. Patients were prescribed a mean 2.5±1.1 antihypertensive drugs. The mean eGFR was 45±29 mL/min/1.73m2. The mean peak systolic velocity was 3.1±1.0 meter/second (Table 1). A computed tomography angiography and a magnetic resonance angiography were performed in 15 and 12 patients, respectively (Fig 1).

**Table 1:** Characteristics of patients with atherosclerotic renal artery disease discussed in the multidisciplinary meeting.

		Total cohort	Medical treatment	Revascularization	*p* value
		(n = 49)	(n = 26)	(n = 23)	
Demographic data					
	Age, years	68.2±12.6	71.4±10.9	64.6 ±13.5	0.057
	Gender, male	35 (71%)	17 (65%)	18 (78%)	0.319
Comorbidities					
	Diabetes	18 (37%)	10 (38%)	8 (35%)	0.790
	Congestive heart failure	25 (51%)	12 (46%)	13 (57%)	0.469
	Atheromatous coronary disease	17 (35%)	9 (35%)	8 (35%)	0.990
Blood pressure					
	Systolic blood pressure, mmHg	146±29	142 ±27	150 ±31	0.333
	Diastolic blood pressure, mmHg	80±16	76 ±15	85 ±17	0.049
	Uncontrolled hypertension (TA>140/90)	29 (59%)	16 (62%)	13 (57%)	0.721
	Resistant hypertension (TA>140/90 with at least 3 antihypertensive drug classes)	12 (24%)	6 (23%)	6 (26%)	0.807
Renal function					
	eGFR (CKD-Epi formula), mL/min/1.73m^2^	45±29	47±28	43±30	0.645
	Proteinuria ≥ 0.5 g/24h	8 (16%)	3 (12%)	5 (22%)	0.448
Medical treatment					
	Any antihypertensive drug	47 (96%)	24 (92%)	23 (100%)	0.491
	ACE inhibitor or ARB	41 (84%)	21 (81%)	20 (87%)	0.706
	Number of antihypertensive drugs in class	2.5±1.1	2.4±1.1	2.6±1.2	0.562
Renal artery disease					
	Bilateral renal-artery disease	6 (12%)	5 (19%)	1 (4%)	0.194
	Stenosis in a solitary functioning kidney	7 (14%)	2 (8%)	5 (22%)	0.230
Radiological parameters (echo-doppler)					
	Peak systolic velocity, m/s	3.1±1.0	2.8±0.9	3.5±1.1	0.019
	Acceleration time, ms	76±53	70±55	82±52	0.463
	Resistive index	0.60±0.11	0.62±0.12	0.58±0.10	0.231

Values are presented as number (%) or mean ± standard deviation

ACE: angiotensin converting enzyme; ARB: angiotensin receptor-blocker; eGFR: estimated glomerular filtration rate.

### Therapeutic decision

Twenty-six patients (53%) were assigned to medical treatment alone and 23 (47%) to medical treatment + revascularization. Among patients with a decision of revascularization, 3 (13%) were assigned to open surgery (Fig 1). For these 3 patients, a computed tomography scan without contrast had been performed before the therapeutic decision was made, and had shown severe atherosclerotic lesions of the aortic wall considered at a very high risk of atheroembolism; moreover, 1 of these patients had a history of cholesterol embolism. Open surgery was a hepato-renal bridge for two patients with a right renal artery disease, and an aorto-aortic bridge with renal artery reimplantation for one patient. All patients were treated in accordance with the decision taken during the meeting.

There was no significant difference between the medical treatment and the revascularization groups except for diastolic blood pressure and peak systolic velocity Doppler data (Table 1).

Respectively 24% (12 patients), 12% (6 patients), and 37% (18 patients) of decisions were not consistent with the 2013 AHA, the 2011 ESC, and the 2017 ESC guidelines.

According to the 2013 AHA guidelines, revascularization was recommended (guideline class I to IIa) for 19 patients (39%), could be considered (guideline class IIb) for 13 patients (27%), and was not recommended for 17 patients (35%; Table 2). Among the 32 patients for whom a revascularization was recommended or suggested (guideline class I to IIb), 14 patients (44%) did not undergo revascularization: for 8 patients (57%), renal artery disease was considered having no hemodynamic impact on parenchymatous vascularization; 5 patients (36%) were considered as contra-indicated based on the presence of one or more arguments against revascularization cited above (age > 85 years, altered general health status, stage 5 chronic kidney disease, and/or kidney length <8 cm); the last patient (7%) had a renal artery disease located on an accessory renal artery. Among the 17 patients for whom revascularization was not recommended or suggested, 5 underwent revascularization (29%): all were young patients (≤65 years of age) without renal insufficiency, with an asymptomatic unilateral stenosis considered to be very tight or even pre-thrombotic based on Doppler and/or computed tomography data.

**Table 2:** Consistency of the therapeutic decision with international guidelines.

	Total cohort	Medical treatment	Revascularization
	(n = 49)	(n = 26)	(n = 23)
2013 AHA guidelines			
- Revascularization recommended	19(39%)	7(27%)	12(52%)
- Revascularization could be considered	13(27%)	7(27%)	6(26%)
- Revascularization not recommended	17(35%)	12(46%)	5(22%)
2011 ESC guidelines			
- Revascularization recommended	0(0%)	0(0%)	0(0%)
- Revascularization could be considered	24(49%)	7(27%)	17(74%)
- Revascularization not recommended	25(51%)	19(73%)	6(26%)
2017 ESC guidelines			
- Revascularization recommended	0(0%)	0(0%)	0(0%)
- Revascularization could be considered	8(16%)	3(12%)	5(22%)
- Revascularization not recommended	41(84%)	23(86%)	18(78%)

Values are presented as number (%)

AHA: American Health Association; ESC: European Society of Cardiology. recommended = based on a guideline class I to IIa; could be considered = based on a guideline class IIb; not recommended = no guideline suggesting revascularization.

According to the 2011 ESC guidelines, revascularization was recommended for no patient (0%), could be considered for 24 patients (49%), and was not recommended for 25 patients (51%; Table 2). Among the 24 patients for whom a revascularization was suggested, 7 (29%) did not undergo revascularization: as above, the reasons were an absence of hemodynamic impact on parenchymatous vascularization (4 patients), a contra-indication (2 patients) or an accessory renal artery disease (1 patient). Among the 25 patients for whom revascularization was not recommended or suggested, 6 underwent revascularization (24%), and these had the same profile as those revascularized despite the 2013 AHA guidelines (≤65 years of age, no renal insufficiency, asymptomatic unilateral stenosis considered to be very tight or pre-thrombotic; 5 similar patients/6).

According to the 2017 ESC guidelines, revascularization was recommended for no patient (0%), could be considered for 8 patients (16%), and was not recommended for 41 patients (84%; Table 2). Among the 8 patients for whom a revascularization was recommended, 3 (37%) did not undergo revascularization: the reasons were a contra-indication (2 patients) or an accessory renal artery disease (1 patient). Among the 41 patients for whom revascularization was not recommended or suggested, 18 underwent revascularization (44%).

### Safety data related to revascularization procedures

The endovascular revascularization procedure failed due to impossible renal artery catheterization for 2 patients. One per-procedure stent thrombosis occurred, followed by thrombo-aspiration and the deployment of a new stent during the same intervention. Three patients developed minor local hematomas without need for a transfusion. No re-stenosis was observed during follow-up. No grade 3 adverse event was reported related to endovascular or open surgical procedures.

### One-year clinical outcomes according to treatment assignment

In the Medical treatment group, at one-year follow-up, patients experienced no significant change in blood pressure values, number of antihypertensive drugs, eGFR and proteinuria (Tables 3 and 4). Among patients with an uncontrolled hypertension at baseline, 31% exhibited a controlled hypertension at one year follow up. Among patients with a resistant hypertension at baseline, 33% did not exhibit a persistent resistant hypertension at one year follow up (Table 4).

**Table 3:** Clinical and renal parameters evolution during one-year follow-up according to assigned treatment.

	Medical treatment (n = 26)		Revascularization (n = 23)	
	Change at 1-year	*p* value*	Change at 1-year	*p* value*
Systolic blood pressure (mmHg)	+6 ± 36	0.439	-23 ± 34	0.007
Diastolic blood pressure (mmHg)	-1 ± 15	0.696	-12 ± 18	0.007
Number of antihypertensive drugs	+0.13 ± 1.14	0.589	-1.00 ± 1.03	0.001
eGFR (mL/min/1.73m^2^)	+2 ± 11	0.485	+4 ± 13	0.151

Values are presented as mean ± standard deviation

eGFR = estimated glomerular filtration rate.

*: Paired t-test or Wilcoxon signed rank test for paired samples

**Table 4:** Status at one year according to assigned treatment.

		Medical treatment		Revascularization	
		n (%)	p*	n (%)	p*
Controlled HT at 1 year (as compared to baseline)			1.000		0.022
	Among patients with controlled HT at baseline	6/10 (60%)		8/10 (80%)	
	Among patients with uncontrolled HT at baseline	5/16 (31%)		11/13 (85%)	
Absence of resistant HT at 1 year (as compared to baseline)			0.289		0.031
	Among patients without resistant HT at baseline	14/20 (70%)		17/17 (100%)	
	Among patients with resistant HT at baseline	2/6 (33%)		6/6 (100%)	
Absence of proteinuria >0.5g/24h (as compared to baseline)			1.000		1.000
	Among patients without proteinuria >0.5g/24h at baseline	23/23 (100%)		17/18 (94%)	
	Among patients with proteinuria >0.5g/24h at baseline	1/3 (33%)		2/5 (40%)	
End stage renal disease		1/26 (4%)		3/23 (13%)	
Death		1/26 (4%)		1/23 (4%)	

HT: Hypertension; Resistant HT: Blood pressure >140/90mmHg in spite of the use of three antihypertensive medications belonging to different drug classes; Uncontrolled HT: Blood pressure >140/90mmHg whatever the antihypertensive treatment.

* McNemar’s test

In the revascularization group, there was a significant improvement of systolic blood pressure (mean decrease, 23±34 mmHg, *p* = 0.007), diastolic blood pressure (mean decrease, 12±18mmHg, *p* = 0.007) and a reduction of the number of antihypertensive drugs (mean decrease, 1.00±1.03 drug/patient, *p* = 0.001; Table 3). Among patients with uncontrolled hypertension at baseline, 85% presented a controlled hypertension at one year follow up. Among patients with a resistant hypertension at baseline, 100% did not exhibit a persistent resistant hypertension at one year follow up. The rates of patients with an uncontrolled hypertension and with a resistant hypertension significantly decreased over time (*p* = 0.022 and 0.031, respectively; Table 4). eGFR and proteinuria did not significantly change (Tables 3 and 4).

## Discussion

In this retrospective cohort study, the conduct of a decision-making process based on a renal artery disease multidisciplinary meeting led to retain the indication of revascularization in approximately 50% of a cohort of patients with a stenosis >60%. Among patients with a decision of revascularization, respectively 24%, 12% and 37% of decisions were not in accordance with the 2013 AHA, 2011 ESC, and 2017 ESC Guidelines. Patients in whom a revascularization was performed exhibited a significant improvement in blood pressure control and a reduction in antihypertensive drugs number with no grade 3 adverse events. Those parameters were not modified among patients assigned to medical treatment alone.

The concept of a multidisciplinary meeting is certainly not original. Its impact on the quality of care has been demonstrated in other fields in which disease complexity and treatment risks are high, such as oncology [20], intensive care [21], and surgery [22]. It is also highly likely that the decision making process for renal artery revascularization in other centres is based on a collective discussion. However, we aimed to conduct a more formalized decision-making process in which all medical charts of patients with atherosclerotic renal artery disease with a peak systolic velocity > 180 cm/s are discussed. Because it was not planned in the study protocol, we could not verify that all the patients diagnosed in our institution with an atherosclerotic renal artery disease had their chart reviewed during the meeting; however, the physicians working in the nephrology and vascular surgery departments reviewed the charts of all the patients they were in charge of. This led to a reduction of selection bias as compared to previous randomized trials [1–3].

Doppler ultrasound was the first-line screening method, in accordance with the 2011 ESC Guidelines [9]. As opposed to other imaging methods, i.e. angiography, computed tomography angiography and magnetic resonance angiography, Doppler ultrasound is non-invasive, does not use iodinated contrast medium and ionizing radiation, is inexpensive, and can be repeated without risk or discomfort [23]. Most evaluations were made by the same angiologist, allowing a reduction of measurements variability. Furthermore, the renal artery peak systolic velocity threshold used herein is reported to have a 95% sensitivity and a 90% specificity for renal artery disease diagnosis [24]. Doppler findings exhibit a strong correlation with invasively measured renal trans-stenotic haemodynamic parameters [15]. It provides various haemodynamic data: direct signs, located at the extrarenal truncular part of the renal artery, and indirect signs, such as a decrease in parenchymatous perfusion [14]. Unfortunately, the renal-aortic ratio [24] and the asymmetry of the intra-renal resistance index could not be analyzed due to missing data. Morphological imaging methods were mostly performed to evaluate the aortic wall or to confirm the severity of the stenosis.

Almost one in 4 therapeutic decisions were not in accordance with the 2013 AHA guidelines [10]. Among patients who underwent revascularization, more than three-quarters of decisions were consistent with these guidelines; the indications were the presence of a bilateral stenosis or a single kidney, a resistant hypertension, a degradation of renal function, or the occurrence of pulmonary edemas. However, 5 patients ≤65 years of age underwent revascularization contrary to the guidelines, in a context of very tight unilateral asymptomatic renal artery disease in order to avoid a renal thrombosis and, thus preserve long-term renal function. We regret that this decision, quite obvious in such a situation, is not supported by any guidelines [9,10,12]. Among patients in the medical treatment group, more than 50% should have had a revascularization indication discussed according to the guidelines. The decision not to perform a revascularization was based on arguments that are not detailed in the guidelines, although the need for a complete patient evaluation is suggested in the 2013 AHA guidelines [10]. Arguments against revascularization were either related to the fact that renal artery disease is a slowly progressive disease (age, general health status) [25], or related to the existence of an advanced and suspected irreversible nephropathy (renal atrophy [23], macroproteinuria [26]). Beyond the consideration of revascularization criteria alone, our approach ensured that the patient’s social circumstances, preferences, comorbidities, and individual risk were taken into account. It is of note that the 2011 and 2017 ESC guidelines [9,12] do not advise renal revascularization with a I or IIa class recommendation in any situation related to atherosclerotic renal artery disease, and that the only indication for revascularization consistent with the 2017 ESC guidelines [12] is sudden pulmonary oedema or recurrent congestive heart failure. In the cohort presented herein, more than three-quarters of revascularization decisions were not in accordance with these guidelines, whose restrictive character could make it difficult to apply to all clinical situations.

As for blood pressure evolution over the one-year follow-up in patients who benefited from revascularization, there was a significant improvement regardless of the endpoint (blood pressure values as continuous or categorical measures, or number of drug classes). The restoration of renal perfusion pressure consecutive to revascularization may have, at least for some patients, reduced the hypersecretion of renin by the juxtaglomerular apparatus, and thus facilitated blood pressure control. The improvement of blood pressure control, although an intermediate criterion, may be of clinical significance, as each difference of 20 mmHg of systolic blood pressure (or 10 mmHg of diastolic blood pressure) is associated with a more than two-fold difference in stroke-related death [27]. As opposed to the findings of most randomized trials [2–4], these results are in accordance with the results from both the CORAL trial [1] and a Cochrane Review [28], that identified a small improvement in blood pressure control and a decrease in the number of antihypertensive medications after renal revascularization [4]. The decrease in the number of drugs may improve patient adherence, especially in this generally old and polymedicated population [29,30], and has a prominent value from an economic point of view.

We did not identify any significant change in eGFR and proteinuria among patients who benefited from revascularization over the one-year follow-up. As demonstrated in a recent study [31], in the setting of ischemic kidney, the contralateral kidney undergoes high blood pressure-induced hyperfiltration; after revascularization and subsequent blood pressure control, the GFR of the contralateral kidney should decrease to its usual value, limiting the possibility of short-term total eGFR improvement [32]. Another possible explanation is that revascularization should enable the introduction or increase in the dose of angiotensin-renin blockers, which can also blunt the improvement of renal function.

No significant improvement in blood pressure values and number of antihypertensive drugs was observed in the medical treatment group. Even if we cannot exclude that this negative result is related to a lack of power or to sampling fluctuations, the change in blood pressure over the one-year follow-up, an increase of 6 mmHg for the systolic blood pressure and a decrease of 1 mmHg for the diastolic blood pressure, had no clinical relevance. Many patients included in the study had benefited from years of specialized medical follow-up prior to the diagnosis of arterial renal disease. In the medical treatment group, patients were already prescribed a mean±SD 2.4±1.1 antihypertensive drugs, and 81% were treated with angiotensin converting enzyme inhibitor or angiotensin receptor-blocker. Thus, room for improvement based on antihypertensive drug optimization may have been quite limited in this context. Moreover, mean blood pressure values in this group were not that high (142/76mmHg) and therefore not all patients needed blood pressure optimization.

There are several limitations to the study. The generalizability of the results may be limited by its single-centre and retrospective nature. The restricted sample size precluded from proper multivariate analyses, and restricted conclusions to significant results. Our methodological approach prevented us from conducting comparisons between patients evolution according to the treatment received: the group assignment resulted from a medical decision and not from a randomization; thus, the two treatment groups were not comparable. Follow-up was restricted to one year and only intermediate criteria were considered. The final decision is not fully transparent because it resulted from physicians’ experience and knowledge; providing an algorithm is therefore impossible since it is a “patient-based” tailored approach. Lastly, albuminuria, which may help in the identification of patients who could benefit from revascularization [26,33,34], was not precisely collected, as parameters related to patients’ preferences and social circumstances.

In conclusion, based on a renal artery disease multidisciplinary meeting, revascularization decisions were in accordance with the 2013 American Health Association and the 2017 European Society of Cardiology guidelines for only 78% and 22% of patients, respectively. Patients on whom we performed a renal artery revascularization exhibited a significant improvement in blood pressure control with few adverse events. Whether or not this beneficial effect results from this decisional process should be formally tested in a prospective randomized controlled trial.
